# A Real‐Word Analysis of the Correlation Between Clinical Efficacy and Predictive Factors of Immune‐Related Adverse Events in Patients With Nonsmall Lung Cancer Treated With Nivolumab Plus Ipilimumab

**DOI:** 10.1002/cam4.70741

**Published:** 2025-04-18

**Authors:** Atsuto Mouri, Hisao Imai, Satoshi Endo, Junichi Nakagawa, Kasumi Tsukamoto, Yuhei Kurata, Ou Yamaguchi, Kenji Masaki, Kosuke Hashimoto, Ayako Shiono, Yu Miura, Kunihiko Kobayashi, Kyoichi Kaira, Hiroshi Kagamu

**Affiliations:** ^1^ Department of Respiratory Medicine, International Medical Center Saitama Medical University Hidaka Japan; ^2^ Division of Respiratory Medicine Gunma Prefectural Cancer Center Ota Japan; ^3^ Department of Respiratory Medicine National Hospital Organization Takasaki General Medical Center Takasaki Japan; ^4^ Department of Pulmonology National Hospital Organization Disaster Medical Center Tokyo Japan; ^5^ Division of Infectious Diseases and Respiratory Medicine, Department of Internal Medicine National Defense Medical College Tokorozawa Japan

**Keywords:** freal‐world, immune‐related adverse events, ipilimumab, nivolumab, nonsmall cell lung cancer

## Abstract

**Background:**

The combination of nivolumab and ipilimumab, which act on different immune checkpoint molecules, is a promising first‐line treatment strategy for advanced nonsmall cell lung cancer (NSCLC). However, real‐world clinical data on this regimen, particularly regarding the relationship between adverse events (AEs) and efficacy, are inadequate.

**Methods:**

This real‐world retrospective study was conducted on patients with advanced or recurrent NSCLC treated using a combination of nivolumab and ipilimumab as a first‐line treatment. We extracted the data of consecutive eligible patients from four institutions in Japan between December 2020 and November 2022.

**Results:**

The study population comprised 184 patients who received nivolumab plus ipilimumab (median follow up period: 13.0 months [0.3–35.0]). In total, 81.0% (*n* = 149) of the patients were men, and the median age was 72.0 years (range: 46–80). The median progression‐free survival (PFS) and overall survival (OS) were 6.6 months (95% confidence interval [CI]: 4.7–8.2) and 17.4 months (95% CI: 11.9–20.4), respectively. Skin disorders, liver dysfunction, thyroid dysfunction, and pneumonitis were the most common adverse events (AEs), with AEs occurring in 154 patients (83.7%). The median PFS in the AE group was longer than that in the non‐AE group (8.2 vs. 2.6 months, *p* < 0.0001). The median OS in the AE group was also better than that in the non‐AE group (19.3 vs. 6.1 months, *p* < 0.0001). Multivariate logistic regression analysis identified smoking history and high PD‐L1 expression as factors related to the incidence of grade 3 and 4 AEs, respectively. The incidence of multiple AEs revealed a significant association with a longer PFS and OS. Skin disorders, adrenal insufficiency, and eosinophilia were the AEs with the greatest impact on survival.

**Conclusions:**

Patients who experienced AEs had significantly longer PFS. Among AEs, the occurrence of skin disorders, adrenal insufficiency, and eosinophilia were likely to prolong PFS and OS.

## 1 Introduction

Nonsmall cell lung cancer (NSCLC) is associated with a high tumor mutation burden similar to that of melanoma. Thus, patients with NSCLC are suitable candidates for receiving immune checkpoint inhibitors (ICIs). Several clinical trials have demonstrated the efficacy of ICIs in the treatment of NSCLC [1, 2, 3]. According to the cancer immunoediting theory, ICI therapy has increased the long‐term progression‐free survival (PFS) and resulted in steady progress in therapeutic approaches [4, 5]. The first‐line treatment strategies for NSCLC include the administration of programmed cell death‐1 (PD‐1) or programmed cell death‐ligand 1 (PD‐L1) inhibitors alone or in combination with platinum‐based chemotherapy [6, 7, 8, 9]. However, the administration of a combination of two immune checkpoint molecules has emerged as the standard of care [10, 11]. Anti‐PD‐(L)1 and anti‐cytotoxic T‐lymphocyte‐associated protein 4 (CTLA‐4) antibodies induce antitumor immune responses via different phases and mechanisms [12, 13]. A combination of nivolumab and ipilimumab has demonstrated favorable 5‐year survival rates, even among patients with a PD‐L1 tumor proportion score (TPS) of < 1%, who respond less frequently to PD‐1/PD‐L1 inhibitors [14]. The CheckMate 227 trial investigating the effects of nivolumab and ipilimumab in patients with metastatic NSCLC revealed a 12‐month PFS rate of 35%–40% [14]. The PFS and objective response rates (ORRs) for all patients treated with nivolumab plus ipilimumab were 4.2 (3.0–5.6) months and 30%, respectively, in a phase III trial [15].

Compared with that associated with the administration of PD‐(L)1 alone, the addition of CTLA‐4 is associated with an increased frequency of immune‐related AEs (irAEs). A meta‐analysis revealed that compared with nivolumab alone, nivolumab plus ipilimumab regimens are associated with a significantly higher risk of incidence of all grades of irAEs, especially higher grades, such as endocrine disruption, pruritus, rash, diarrhea, and colitis [16, 17]. Analyses restricted to NSCLC have reported similar results [18, 19]. These drug regimens are associated with higher clinical efficacy; nevertheless, the risk of incidence of irAEs must be considered carefully. The affected organs and frequency of irAEs vary according to the type of ICI administered [20]. An analysis of AE rates and treatment‐related mortality was performed in the LIGHTNING study, a multicentre observational study that provided meaningful real‐world data on nivolumab plus ipilimumab treatment [21]. The frequency of treatment‐related AEs of grade 3 or higher among the patients who received nivolumab plus ipilimumab with and without platinum doublet chemotherapy was 44.3% and 27.0%, respectively. Notably, five treatment‐related deaths (3.5%), comprising four cases of pneumonia (2.8%) and one case of sepsis (0.7%), were reported in this study. However, data regarding the incidence of low‐grade AEs and the association between the incidence of AEs and efficacy were lacking in this study.

Information regarding the safety and efficacy of nivolumab plus ipilimumab in routine clinical practice is lacking. irAEs, which may occur secondary to ICI treatment, may represent a driven immune response. Most cases of irAEs are mild; however, severe cases have been reported. Some reports have suggested that irAEs associated with anti‐PD‐(L)1 antibody agents alone or in combination with anticancer agents may be correlated with efficacy [22, 23, 24, 25, 26, 27]. Overall survival (OS) of the patients with NSCLC and thyroid dysfunction who were treated with pembrolizumab was significantly longer than that of patients who were not (hazard ratio 0.29, 95% confidence interval [CI] 0.09–0.94, *p* = 0.04) [28]. A subgroup analysis of the incidence of irAEs and the efficacy of ICIs revealed that thyroid dysfunction and gastrointestinal, cutaneous, or endocrine involvement were particularly associated with better PFS and OS [29]. Some irAEs associated with PD‐(L)1 therapy, such as thyroid dysfunction and skin disorders, may prolong survival. In contrast, other irAEs, such as pneumonitis, may not be correlated with or worsen prognosis [30, 31]. However, the relationship between the incidence of irAEs and the efficacy of anti‐PD‐1 along with anti‐CTLA‐4 antibody therapy in the management of NSCLC remains underexplored.

Therefore, this retrospective study aimed to determine the correlation between the efficacy and safety of nivolumab plus ipilimumab and to identify the associated clinical characteristics.

## 2 Methods

### 2.1 Study Design and Patients

A database created using an original standardized template was used in this multicentre retrospective study. Data regarding the following aspects of patients treated with nivolumab plus ipilimumab at four participating institutions in Japan were extracted: baseline demographic characteristics, histology, staging, clinical and laboratory data, metastatic lesions, medication, body mass index (BMI), and surgical or radiological history. The case accrual period was defined as the period between December 2020 and November 2022, comprising a 1‐year observation period from the last enrolled patient to evaluate the efficacy and safety of this treatment. The date of the last follow‐up visit was 31 October 2023. Patients with histologically or cytologically diagnosed advanced or relapsed chemotherapy‐naïve NSCLC were eligible for inclusion in this study. Patients who had received chemotherapy before commencing nivolumab plus ipilimumab therapy were excluded from this study. In addition, patients who had received nivolumab and ipilimumab with platinum doublet chemotherapy were also excluded. However, those who had received cytotoxic anticancer agents with curative intent and had undergone irradiation or surgery were included.

The clinical stages were classified according to the eighth edition of the TNM classification system [32]. The identification of the driver genes and PD‐L1 TPS was not mandatory; however, testing was performed, and the patient population mainly comprised those who were negative for epidermal growth factor receptor, anaplastic lymphoma kinase, and ROS proto‐oncogene 1 rearrangement. The PD‐L1 levels were measured using the Pharm Dx 22C3 PD‐L1 assay (Agilent, Santa Clara, CA, USA); however, untested cases were also included. Cases wherein the pretreatment demographics, efficacy, and safety could not be assessed and patients deemed ineligible for enrollment by attending physicians at each site were excluded.

This study was approved by the Clinical Research Review Board of Saitama Medical University (2023‐080) and adhered to the principles of the Declaration of Helsinki. The requirement for obtaining informed consent from the participants was waived owing to the retrospective nature of the study.

### 2.2 Treatment and Adverse Events

Nivolumab was administered intravenously at a dose of 360 mg/day once every 3 weeks or 240 mg/day once every 2 weeks. Ipilimumab was administered intravenously at a dose of 1 mg/kg once every 6 weeks until disease progression, unacceptable toxicity, or discontinuation, as determined by the treating physician.

The main irAEs were thyroiditis, adrenal insufficiency, pneumonitis, skin disorders, diarrhea, liver dysfunction, and myositis. Other events deemed irAEs by the attending physician were also collected. The study did not include concomitant cytotoxic anticancer agents. Any event occurring after nivolumab and ipilimumab administration, whether posttreatment or until the next subsequent drug administration, was considered an irAE. The Common Terminology Criteria for Adverse Events version 5.0 was used to assess and grade toxicity. The Response Evaluation Criteria in Solid Tumors version 1.1 was used by the attending physician and radiologist to assess treatment efficacy [33]. Clinical data were anonymised and stored securely as password‐protected case report forms by the local researchers. The efficacy of nivolumab plus ipilimumab in terms of survival and ORR was compared after a fixed observation period based on the presence and grading of AEs. In‐depth analyses were performed to identify the background factors associated with AEs and the specific AEs linked to better outcomes.

### 2.3 Statistical Analyses

PFS from the first administration of nivolumab and ipilimumab to radiographic or clinical progression or death from any cause, and OS from the first administration of nivolumab and ipilimumab to death or date of last follow‐up were estimated using the Kaplan–Meier method and compared using the log‐rank test. Hazard ratios were estimated through Cox regression analysis. The median PFS and OS with 95% CI were calculated. Subgroup analyses of the effectiveness outcomes were conducted based on patient characteristics, including PD‐L1 expression and AEs. As a precautionary measure, an additional analysis was conducted to evaluate the difference in PFS and OS curves, as estimated by the Kaplan–Meier method, based on the presence or absence of irAE in the 6‐week landmark analysis using the log‐rank test [24]. The background characteristics of the groups with and without AEs were compared using the chi‐square test or Fisher's exact test. The baseline characteristic subgroups and specific factors associated with the incidence of irAEs were assessed using univariate and multivariate analyses with logistic regression. Detailed AEs with the greatest prognostic impact were analyzed using logistic regression analysis. Statistical significance was set at *p* < 0.05. All statistical analyses were performed using Prism 9 software (GraphPad Software, San Diego, CA, USA) and JMP 14.0 (SAS Institute Inc., Cary, North Carolina, USA).

## 3 Results

### 3.1 Characteristics of Patients and Treatment Information

The median follow‐up duration of the 184 patients included in the final analysis at the data cutoff point (December 31, 2022) was 13.0 months (range: 0.3–35.0). Table 1 summarizes the patient characteristics according to PD‐L1 TPS expression. The median age of the patients was 72 years (range, 46–86), and 81.0% (*n* = 149) participants were men. The histological types comprised squamous cell carcinoma (*n* = 55) and adenocarcinoma (*n* = 104). The Eastern Cooperative Oncology Group performance status (PS) was 0 or 1 in 83.7% (*n* = 154) of patients. The majority of patients had stage IV disease (*n* = 120); however, 33 patients with postoperative recurrence were included. The TPS expression based on PD‐L1 immunohistochemistry was < 1%, 1%–49%, and ≥ 50% in 45.7% (*n* = 84), 38.6% (*n* = 71), and 9.8% (*n* = 18) of patients, respectively. Among the patients who were tested, those who were negative for *EGFR* and *ALK* but had exon20 insertion mutations, *HER2* mutations, or *KRAS* mutations were selected. The bone was the most common site of metastasis prior to treatment (*n* = 59), followed by effusion (*n* = 45), the brain (*n* = 29), and liver (*n* = 20). The median BMI was < 22 and > 22 kg/m^2^ in 90 and 94, respectively. The Glasgow prognostic score (GPS) was 0/1 and 2 in 68.5% (*n* = 126) and 31.5% (*n* = 58) of patients, respectively. When the neutrophil‐to‐lymphocyte ratio (NLR) cutoff value was set as 5, 68, and 116 patients had higher and lower NLR values, respectively. Table 1 presents the number of patients with platelet‐to‐lymphocyte ratio (PLR) and prognostic nutritional index (PNI) values lower and higher than the median based on the overall median value. During the observation period, 52 patients (28.3%) were transferred to second‐line treatment, 96 patients (52.2%) were not, and 36 patients (19.6%) were considered for future treatment.

**TABLE 1:** Characteristics of the 184 patients.

Patient characteristics	Total	PD‐L1 ≧ 50%	PD‐L1 1%–49%	PD‐L1 < 1%	Unknown
	*n*, 184 (%)	*n*, 18 (%)	*n*, 71 (%)	*n*, 84 (%)	*n*, 11 (%)
Age					
Median (range), years	72.0 (46–86)	69.5 (46–83)	72.0 (48–86)	72.0 (47–85)	70.0 (54–77)
Sex					
Male	149 (81.0)	16 (88.9)	58 (81.7)	65 (77.4)	10 (90.9)
Female	35 (19.0)	2 (11.1)	13 (18.3)	19 (22.6)	1 (9.1)
History of smoking					
Yes	164 (89.1)	18 (100)	65 (91.5)	70 (83.3)	11 (100)
No	20 (10.9)	0	6 (8.5)	14 (16.7)	0
Histologic type					
SQ	55 (29.9)	8 (44.4)	26 (36.6)	18 (21.4)	3 (27.3)
AD	104 (56.5)	9 (50)	38 (53.5)	53 (63.1)	4 (36.4)
Others	25 (13.6)	1 (5.6)	7 (9.9)	13 (15.5)	4 (36.4)
ECOG‐PS					
0	61 (33.2)	3 (16.7)	30 (42.3)	26 (31.0)	2 (18.2)
1	93 (50.5)	11 (61.1)	29 (40.8)	45 (53.6)	8 (72.7)
≧ 2	30 (16.3)	4 (22.2)	12 (16.9)	13 (15.5)	1 (9.1)
Clinical stage					
III	11 (6.0)	1 (5.6)	5 (7.0)	5 (6.0)	0
IV	120 (65.2)	12 (66.7)	41 (57.7)	59 (70.2)	8 (72.7)
Rec post‐surgery^a^	33 (17.9)	2 (11.1)	15 (21.1)	15 (17.9)	1 (9.1)
Rec post‐curative radiation^a^	23 (12.5)	3 (16.7)	10 (14.1)	8 (9.5)	2 (18.2)
Metastatic site^b^					
Brain	29 (15.8)	4 (22.2)	14 (19.7)	11 (13.1)	0
Liver	20 (10.9)	3 (16.7)	12 (16.9)	5 (6.0)	0
Bone	59 (32.1)	7 (38.9)	25 (35.2)	23 (27.4)	4 (36.4)
Effusion	45 (24.5)	3 (16.7)	15 (21.1)	23 (27.4)	4 (36.4)
BMI					
< 22	90 (48.9)	7 (38.9)	37 (52.1)	42 (50.0)	4 (36.4)
≧ 22	94 (51.0)	11 (61.1)	34 (47.9)	42 (50.0)	7 (63.6)
GPS					
0/1	126 (68.5)	10 (55.6)	49 (69.0)	60 (71.4)	7 (63.6)
2	58 (31.5)	8 (44.4)	22 (31.0)	24 (28.6)	4 (36.4)
NLR					
< 5	116 (63.0)	8 (44.4)	41 (57.7)	59 (70.2)	8 (72.7)
≧ 5	68 (36.9)	10 (55.6)	30 (42.3)	25 (29.8)	3 (27.3)
PLR					
< 242	93 (50.5)	9 (50.0)	31 (43.7)	46 (54.8)	7 (63.6)
≧ 243	91 (49.5)	9 (50.0)	40 (56.3)	38 (45.2)	4 (36.4)
PNI					
< 64	93 (50.5)	10 (55.6)	42 (59.2)	37 (44.0)	4 (36.4)
≧ 64	91 (49.5)	8 (44.4)	29 (40.8)	47 (56.0)	7 (63.6)
Transition to second‐line treatment					
Yes	52 (28.3)	5 (27.8)	24 (33.8)	19 (22.6)	4 (36.4)
No	96 (52.2)	9 (50.0)	33 (44.6)	47 (56.0)	7 (63.6)
Not yet	36 (19.6)	4 (22.2)	14 (19.7)	18 (21.4)	0

Abbreviations: AD, adenocarcinoma; BMI, body mass index; ECOG‐PS, Eastern Cooperative Oncology Group performance status; GPS, Glasgow prognostic score; NLR, neutrophil‐to‐lymphocyte ratio; PD‐L1, programmed death‐ligand 1; PLR, platelet‐lymphocyte ratio; PNI, prognostic nutritional index; Rec, recurrence; SQ, squamous cell carcinoma; TPS, tumor proportion score.

^a^ Three patients who relapsed after surgery and were treated with curative radiation were included.

^b^ Patients with multiple metastatic sites were included in the study.

### 3.2 Safety

Table 2 presents the treatment safety profiles assessed using the common terminology criteria for AEs. AEs of all grades occurred in 83.7% (*n* = 154) of patients. Skin disorders (37.5%), including abnormal skin events such as pruritus, erythema, and rash; liver dysfunction (29.9%); hyperthyroidism or hypothyroidism (21.7%); pneumonitis (19.6%); adrenal insufficiency (17.4%); diarrhea (16.3%); and increased creatinine (15.2%) were the most common AEs. AEs of grade 3 or higher occurred in 39.1% (*n* = 72) of patients, with pneumonia being the most common (10.9%). This was followed by adrenal insufficiency (9.2%), liver dysfunction (8.2%), diarrhea (6.5%), skin disorders (4.3%), and myositis (1.1%). AE leading to interruption and discontinuation occurred in 23.9% and 54.3% of patients, respectively. Treatment‐related death due to pneumonitis occurred in one case. Unexpected AEs were not observed.

**TABLE 2:** Adverse events associated with nivolumab plus ipilimumab (%).

Adverse event leading to interruption *n* (%)	44 (23.9)
Adverse event leading to discontinuation *n* (%)	100 (54.3)
Adverse event leading to death *n* (%)	1 (0.5) Pneumonitis

Variable	All grade	≥ Grade 3
	*n* (%)	*n* (%)
Any adverse events	154 (83.7)	72 (39.1)
Skin disorder^a^	69 (37.5)	8 (4.3)
Liver dysfunction^b^	55 (29.9)	15 (8.2)
Hyper or hypothyroidism	40 (21.7)	0
Pneumonitis	36 (19.6)	20 (10.9)
Adrenal insufficiency	32 (17.4)	17 (9.2)
Diarrhea	30 (16.3)	12 (6.5)
Increase in Cr levels	28 (15.2)	0
Increase inγ‐GTP levels	19 (10.3)	0
Eosinophilia	17 (9.2)	0
Fever	14 (7.6)	0
Increase in ALP levels	12 (6.5)	0
Arthritis	12 (6.5)	0
Myositis^c^	12 (6.5)	2 (1.1)
Stomatitis	5 (2.7)	0
Increase in amylase levels	5 (2.7)	0
Decrease in PLT count	5 (2.7)	0
Dizziness	2 (1.1)	0
Type 1 diabetes	2 (1.1)	1 (0.5)
Peripheral sensory neuropathy	1 (0.5)	0
Encephalopathy	1 (0.5)	1 (0.5)
Decrease in WBC count	1 (0.5)	0

Abbreviations: ALP, alkaline phosphatase; Cr, creatinine; PLT, platelet; WBC, white blood cell; γ‐GTP, γ‐glutamyl transferase.

^a^ Skin disorders included abnormal skin events such as pruritus, erythema, and rash.

^b^ Liver dysfunction included elevation of aspartate aminotransferase and alanine aminotransferase levels.

^c^ Myositis also includes symptomless CK elevations.

Table 3 presents the background factors of patients with and without all grades of AEs and those with and without AEs of grade 3 or higher. Analysis of the factors involved in the development of AEs revealed that squamous cell carcinoma, PS 0/1, PD‐L1 ≥ 50%, and PNI ≥ 64 differed significantly for the incidence of all grades of AEs in univariate analysis. However, only PS 0/1 and PD‐L1 ≥ 50% were identified as significant independent factors in multivariate analysis. A history of smoking, no history of radical irradiation, and PD‐L1 ≥ 50 differed significantly for the incidence of AEs of grade 3 or higher in univariate analysis. However, only a history of smoking and PD‐L1 ≥ 50% were identified as significant independent factors in multivariate analysis (Table 4).

**TABLE 3:** Baseline characteristics of the 184 patients according to the presence of adverse events.

Patient characteristics	Total *n*, 184 (%) (range)	AE (+)	AE (−)	*p*	≧ G3 AE (+)	≧ G3 AE (−)	*p*
		*N* = 154	*N* = 30		*N* = 72	*N* = 112	
Age							
Median (range), years	72.0 (46–86)	72.0 (46–86)	72.0 (48–82)	—	71.0 (46–86)	72.0 (48–82)	—
< 75	127 (69.0)	106 (68.8)	21 (70)	0.90	52 (72.2)	75 (67.0)	0.52
≧ 75	57 (31.0)	48 (31.2)	9 (30)		20 (27.8)	37 (33.0)	
Sex							
Male	149 (81.0)	128 (83.1)	21 (70)	0.09	59 (81.9)	90 (80.4)	0.85
Female	35 (19.0)	26 (16.8)	9 (30)		13 (18.1)	22 (19.6)	
Smoking history							
Yes	164 (89.1)	139 (90.3)	25 (83.3)	0.26	69 (95.8)	95 (84.8)	0.03*
No	20 (10.9)	15 (9.7)	5 (16.7)		3 (4.2)	17 (15.2)	
Histologic type							
Squamous	55 (29.9)	51 (33.1)	4 (13.3)	0.06	23 (31.9)	32 (28.6)	0.85
Adenocarcinoma	104 (56.5)	84 (54.5)	20 (66.7)		39 (54.2)	65 (58.0)	
Others	25 (13.6)	19 (12.)	6 (20)		10 (13.9)	15 (13.4)	
ECOG‐PS							
0	61 (33.2)	56 (36.4)	5 (16.7)	0.03*	21 (29.2)	40 (35.7)	0.38
1	93 (50.5)	77 (50)	16 (53.3)		41 (56.9)	52 (46.4)	
≧ 2	30 (16.3)	21 (13.6)	9 (30)		10 (13.9)	20 (17.9)	
Clinical stage							
III	11 (6.0)	10 (6.5)	1 (3.3)	0.03*, ^b^	4 (5.6)	7 (6.3)	0.15
IV	120 (65.2)	94 (61.0)	26 (86.7)		51 (70.8)	69 (61.6)	
Rec postsurgery^a^	33 (17.9)	31 (20.1)	2 (6.7)		13 (18.1)	20 (17.9)	
Rec postcurative radiation^a^	23 (12.5)	21 (13.6)	2 (6.7)		4 (5.6)	19 (17.0)	
PD‐L1 (%) expression							
< 1	84 (45.7)	69 (44.8)	15 (50)	0.16	33 (45.8)	51 (45.5)	0.04*
1–49	71 (38.6)	59 (38.3)	12 (40)		24 (33.3)	47 (42.0)	
≥ 50	18 (9.8)	18 (11.7)	0		12 (16.7)	6 (5.4)	
Unknown	11 (6.0)	8 (5.2)	3 (10)		3 (4.2)	8 (7.1)	
< 1%	84 (45.7)	69 (44.8)	15 (50)	0.42	33 (45.8)	51 (45.5)	0.88
≧ 1%	89 (48.4)	77 (50)	12 (40)		36 (50)	53 (47.3)	
Unknown	11 (6.0)	8 (5.2)	3 (10)		3 (4.2)	8 (7.1)	
< 50%	155 (84.2)	128 (83.1)	27 (90)	0.08	57 (79.2)	98 (87.5)	0.02*
≧ 50%	18 (9.8)	18 (11.7)	0		12 (16.7)	6 (5.4)	
Unknown	11 (6.0)	8 (5.2)	3 (10)		3 (4.2)	8 (7.1)	
Metastatic site^c^							
Brain	29 (15.8)	27 (17.5)	2 (6.7)	0.18	12 (16.7)	17 (15.2)	0.83
Liver	20 (10.9)	15 (9.7)	5 (16.7)	0.33	7 (9.7)	13 (11.6)	0.81
Bone	59 (32.1)	43 (27.9)	16 (53.3)	0.01*	22 (30.6)	37 (33.0)	0.75
Effusion	45 (24.5)	35 (22.7)	10 (33.3)	0.25	19 (26.4)	26 (23.2)	0.73
BMI							
< 22	90 (48.9)	73 (47.4)	17 (56.7)	0.43	36 (50)	54 (48.2)	0.88
≧ 22	94 (51.0)	81 (52.6)	13 (43.3)		36 (50)	58 (51.8)	
GPS							
0/1	126 (68.5)	109 (70.8)	17 (56.7)	0.14	50 (69.5)	76 (67.9)	0.87
2	58 (31.5)	45 (27.3)	13 (43.3)		22 (30.6)	36 (32.1)	
NLR							
< 5	116 (63.0)	98 (63.6)	18 (60)	0.84	49 (68.1)	67 (59.8)	0.28
≧ 5	68 (36.9)	56 (36.4)	12 (20)		23 (31.9)	45 (40.2)	
PLR							
< 242	93 (50.5)	81 (52.6)	12 (40)	0.23	42 (58.3)	51 (45.5)	0.10
≧ 243	91 (49.5)	73 (47.4)	18 (60)		30 (41.7)	61 (54.5)	
PNI							
< 64	93 (50.5)	73 (47.4)	20 (66.7)	0.07	31 (43.1)	62 (55.4)	0.13
≧ 64	91 (49.5)	81 (52.6)	10 (33.3)		41 (56.9)	50 (44.6)	

Abbreviations: AD, adenocarcinoma; AE, adverse event; BMI, body mass index; ECOG‐PS, Eastern Cooperative Oncology Group performance status; G3, grade 3; GPS, Glasgow prognostic score; NLR, neutrophil‐to‐lymphocyte ratio; NOS, not otherwise specified; PD‐L1, programmed death ligand 1; PLR, platelet‐lymphocyte ratio; PNI, prognostic nutritional index; Rec, recurrence; SQ, squamous cell carcinoma.

^a^ Three patients who relapsed after surgery and were treated with curative radiation were included.

^b^ Significant difference tests were performed between the two groups for stage III/IV disease and postoperative recurrence or recurrence after radical irradiation.

^c^ Patients with multiple metastatic sites were included in this study.

* *p* < 0.05.

**TABLE 4:** Logistic regression analysis of the factors associated with adverse events.

		All grades of AE						≧ Grade 3 AE					
Variables		Univariate			Multivariate			Univariate			Multivariate		
		Odds ratio	95% CI	*p*	Odds ratio	95% CI	*p*	Odds ratio	95% CI	*p*	Odds ratio	95% CI	*p*
Age	≧ 75	1.26	0.51–3.39	0.63	1.18	0.45–3.36	0.74	0.78	0.40–1.48	0.45	0.73	0.36–1.45	0.38
Sex	Male	0.46	0.81–5.34	0.12				1.11	0.52–2.42	0.79			
History of smoking	Yes	1.61	0.43–4.90	0.44	1.23	0.31–4.07	0.74	4.12	1.31–18.1	0.01*	4.04	1.26–18.09	0.02*
Histologic type	SQ	3.76	1.23–16.4	0.02*	4.51	1.37–20.9	0.01*	1.17	0.61–2.23	0.63			
	AD	0.65	0.26–1.51	0.32				0.85	0.47–1.55	0.61			
ECOG‐PS	≧ 2	0.29	0.12–0.75	0.01*	0.27	0.08–0.87	0.03*	0.74	0.31–1.65	0.47	0.55	0.20–1.45	0.23
Clinical stage	Rec postsurgery^a^	2.93	0.81–18.8	0.11				1.18	0.54–2.52	0.67			
	Rec postcurative radiation^a^	1.84	0.49–12.0	0.40				0.29	0.08–0.81	0.02*			
PD‐L1 expression	≧ 1	1.12	0.49–2.62	0.78				1.09	0.60–1.98	0.77			
	≧ 50	5,494,998.3	1.58–NA	0.02*	5,494,998.3	1.65–NA	0.02*	3.53	1.30–10.6	0.01*	3.89	1.35–12.7	0.01*
BMI	< 18.5	0.40	0.15–1.22	0.10				0.51	0.17–1.29	0.16			
	≧ 25	0.79	0.29–2.54	0.67				0.51	0.20–1.18	0.12			
GPS	2	0.48	0.21–1.13	0.09	0.68	0.24–2.08	0.49	0.92	0.49–1.75	0.82	1.15	0.52–2.54	0.73
NLR	≧ 5	0.77	0.33–1.83	0.54	0.88	0.33–2.42	0.80	0.70	0.37–1.30	0.26	0.73	0.34–1.45	0.73
PLR	≧ 243	0.47	0.19–1.09	0.08				0.60	0.33–1.08	0.09			
PNI	≧ 64	2.49	1.05–6.38	0.04*				1.64	0.91–3.00	0.10			

Abbreviations: AD, adenocarcinoma; BMI, body mass index; ECOG‐PS, Eastern Cooperative Oncology Group performance status; GPS, Glasgow prognostic score; NLR, neutrophil‐to‐lymphocyte ratio; NOS, not otherwise specified; PD‐L1, programmed death ligand 1; PLR, platelet‐to‐lymphocyte ratio; PNI, prognostic nutritional index: NA, Not available; Rec, recurrence; SQ, squamous cell carcinoma.

^a^ Three patients who relapsed after surgery and were treated with curative radiation were included.

* *p* < 0.05.

### 3.3 Efficacy

**FIGURE 1:**
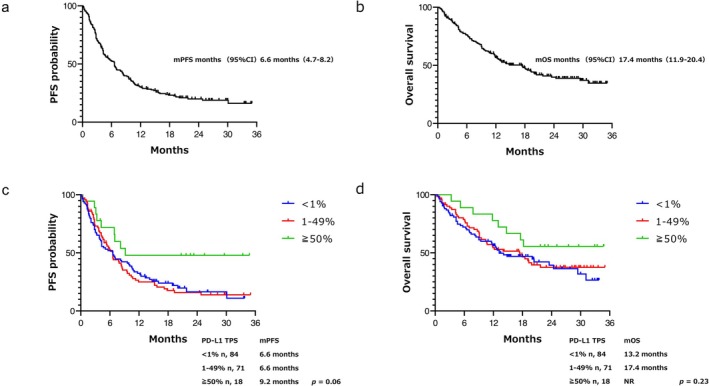
Kaplan–Meier survival curves for PFS (a) and OS (b). Kaplan–Meier curves for PFS (c) and OS (d) described separately for each staining percentage of PD‐L1. CI, confidence interval; NR, not reached; OS, overall survival; PD‐L1, programmed death‐ligand 1; PFS, progression‐free survival.

The median PFS and OS were 6.6 months (95% CI: 4.7–8.2) and 17.4 months (95% CI: 11.9–20.4), respectively (Figure 1a,b). The 6‐ and 12‐month PFS rates were 52.7% (95% CI: 45.5–59.8) and 30.2% (95% CI: 23.7–36.9), respectively. The 6‐ and 12‐month OS rates were 76.3% (95% CI: 69.4–81.7) and 56.8% (95% CI: 49.6–63.7), respectively. In addition, the 173 patients who had undergone PD‐L1 TPS staining were divided into three groups: the PD‐L1 < 1%, 1%–49%, and ≥ 50% groups. The PFS in the PD‐L1 < 1%, 1%–49%, and ≥ 50% groups was 6.6 months (95% CI: 4.1–10.1), 6.6 months (95% CI: 4.6–8.4), and 9.2 months (95% CI: 4.2–not reached), respectively. The OS in the PD‐L1 < 1%, 1%–49%, and ≥ 50% groups was 13.2 months (95% CI: 9.0–24.4), 17.4 months (95% CI: 9.5–21.7), and not reached (95% CI: 13.0–not reached), respectively (Figure 1c,d). Complete response was observed in one patient, whereas partial response was observed in 61 patients. Stable disease was observed in 59 patients, whereas progressive disease was observed in nine patients. The ORR and disease control rate were 32.1% (95% CI: 27.3–40.8) and 65.2% (95% CI: 58.6–72.2), respectively (Figure S1 and Table S1). The ORR in the PD‐L1 < 1%, 1%–49%, and ≥ 50% groups was 31.0% (95% CI: 22.0–41.5), 29.6% (95% CI: 20.2–41.1), and 50% (95% CI: 29.0–71.0), respectively.

### 3.4 Relationship Between Adverse Events and Efficacy

**FIGURE 2:**
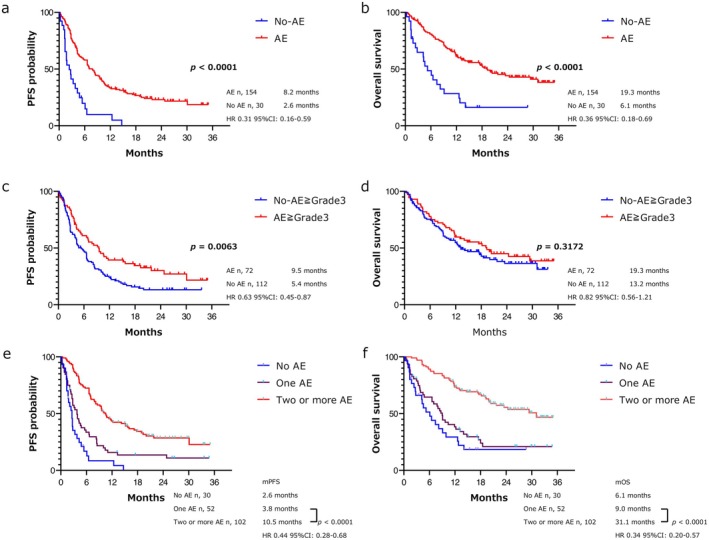
Kaplan–Meier curves for PFS (a) and OS (b) for patients with and without adverse events of all grades. Kaplan–Meier curves for PFS (c) and OS (d) for groups with and without adverse events of grade 3 or higher. Kaplan–Meier curves for PFS (e) and OS (f) for patients with multiple adverse events, single adverse event. and no adverse events. AE, adverse event; C, confidence interval; NR, not reached; PFS, progression‐free survival; OS, overall survival; PD‐L1, programmed death‐ligand 1.

The PFS and OS of the patients with any grade AEs were 8.2 and 19.3 months, respectively, when categorized according to the presence of AEs. The PFS and OS of the patients without AEs were 2.6 and 6.1 months, respectively (PFS *p* < 0.0001, HR: 0.31, 95% CI: 0.16–0.59; OS *p* < 0.0001, HR: 0.36, 95% CI: 0.18–0.69) (Figure 2a,b). Seventy‐two patients experienced AEs of grade 3 or higher, whereas 112 patients experienced AEs of lower grades. The PFS in the high‐ and low‐grade AE groups was 9.5 and 5.4 months, respectively (*p* = 0.0063, HR: 0.63, 95% CI: 0.45–0.87). The OS in the high‐ and low‐grade AE groups was 19.3 and 13.2 months, respectively (*p* = 0.32, HR: 0.82, 95% CI: 0.56–1.21) (Figure 2c,d). The ORR in the groups with all‐grade AEs and without AEs was 37.0% and 6.7%, respectively (*p* = 0.015). In a 6‐week landmark analysis of 165 patients, the PFS and OS in the group with all‐grade AEs were 8.5 and 21.7 months, respectively, while those without all‐grade AEs were 2.8 and 8.2 months, respectively, both significantly longer (*p* < 0.0001) (Figure S3). The PFS and OS in the group with grade 3 or higher AEs were 9.9 and 20.4 months, respectively, while the PFS and OS in the group without grade 3 or higher AEs were 6.6 and 17.6 months, showing a significant difference only in PFS (*p* = 0.007) (Figure S3). In addition, A subgroup analysis comparing nivolumab plus ipilimumab with and without all‐grade AEs by smoking status showed significant differences in both PFS and OS (Figure S4). The ORR in the groups with and without AEs of grade 3 or higher differed significantly (44.4% and 24.1%, respectively; *p* = 0.01) (Figure S1). There was a significant difference in ORR between patients with and without all‐grade AEs, as well as between those with and without grade 3 or higher AEs. Additionally, a significant difference was observed in the number of progression‐free patients at 6 and 12 months between the two groups based on the presence or absence of all‐grade AEs (Table S2). Comparison between the survival of patients with multiple AEs and those with only one AE revealed a significant difference (PFS: 3.8 and 10.5 months, respectively; *p* < 0.0001; HR: 0.44; 95% CI: 0.28–0.68) (OS: 9.0 and 31.1 months, respectively; *p* < 0.0001; HR: 0.34; 95% CI: 0.20–0.57), with the survival in the group of patients with multiple AEs being significantly longer (Figure 2e,f). Furthermore, the survival in the group of patients with ≥ 3 AEs was significantly longer than that in the group of patients with ≤ 2 AEs. Similarly, the survival in the group of patients with ≥ 4 AEs was significantly longer than that in the group of patients with ≤ 3 AEs (Figure S2).

A subset analysis of irAEs revealed skin disorders, adrenal insufficiency, eosinophilia, diarrhea, and fever as items significantly prolonging PFS. Skin disorders, adrenal insufficiency, and eosinophilia were identified as independent factors prolonging PFS in the multivariate analysis. Skin disorders, adrenal insufficiency, diarrhea, and eosinophilia were identified as items significantly prolonging OS in the univariate. However, only skin disorders, adrenal insufficiency, and eosinophilia were identified as independent factors prolonging OS in the multivariate analysis (Table 5).

**TABLE 5:** Univariate and multivariate analyses of adverse events associated with (a) PFS and (b) OS.

Factor		(a) PFS	Univariate			Multivariate			(b) OS	Univariate			Multivariate		
		Median	HR	95% CI	*p*	HR	95% CI	*p*	Median	HR	95% CI	*p*	HR	95% CI	*p*
Skin disorder^a^	Yes/No	11.3/4.6	0.49	0.34–0.69	< 0.01***	0.55	0.38–0.78	< 0.01***	31.1/9.9	0.41	0.26–0.63	< 0.01***	0.47	0.30–0.71	< 0.01***
Liver dysfunction^b^	Yes/No	9.5/5.6	0.74	0.51–1.05	0.10				20.4/12.6	0.65	0.41–1.00	0.06			
Hyper or hypothyroidism	Yes/No	9.4/5.7	0.27	0.53–1.18	0.28				21.7/13.4	0.66	0.40–1.06	0.09			
Pneumonitis	Yes/No	7.8/6.6	0.79	0.50–1.19	0.27				19.1/14.8	0.96	0.58–1.51	0.85			
Adrenal insufficiency	Yes/No	15.7/5.3	0.35	0.18–0.63	< 0.01**	0.58	0.36–0.91	0.02	NR/13.0	0.37	0.19–0.67	< 0.01*	0.44	0.22–0.79	< 0.01**
Diarrhea	Yes/No	9.2/5.7	0.56	0.33–0.88	0.02*				NR/13.1	0.54	0.28–0.95	0.04*			
Increase in Cr levels	Yes/No	9.8/5.4	0.61	0.37–0.97	0.05				23.4/14.0	0.64	0.34–1.10	0.13			
Biliary system abnormalities^c^	Yes/No	6.6/6.6	1.09	0.68–1.88	0.73				18.0/13.0	1.22	0.69–2.03	0.45			
Eosinophilia	Yes/No	NR/5.9	0.32	0.13–0.64	< 0.01**	0.40	0.17–0.81	< 0.01**	NR/13.0	0.20	0.05–0.54	< 0.01*	0.27	0.07–0.73	< 0.01**
Fever	Yes/No	20/5.9	0.46	0.16–1.01	0.01*				NR/14.1	0.46	0.16–1.01	0.08			
Endocrine^d^	Yes/No	9.2/4.8	0.73	0.50–1.03	0.08				20.4/12.8	0.65	0.41–0.99	0.05			

Abbreviations: BMI, body mass index; CI, confidence interval; Cr, creatinine; ECOG‐PS, Eastern Cooperative Oncology Group performance status; GPS, Glasgow prognostic score; HR, hazard ratio; NLR, neutrophil‐to‐lymphocyte ratio; NR, not reached; PD‐L1, programmed death ligand 1; Rec, recurrence; Sq, squamous carcinoma.

^a^ Skin disorders here included abnormal skin events such as pruritus, erythema, and rash.

^b^ Liver dysfunction here included elevation of aspartate aminotransferase and alanine aminotransferase levels.

^c^ Biliary system abnormalities include elevations in gamma‐glutamyl transferase, alkaline phosphatase, and amylase levels.

^d^ Endocrine included cases of either thyroid dysfunction or adrenal dysfunction.

* *p* < 0.05.

** *p* < 0.01.

*** *p* < 0.001.

## 4 Discussion

The present study demonstrated that the incidence of AEs exhibited an association with better survival in patients with NSCLC treated with nivolumab plus ipilimumab. Furthermore, skin disorders, adrenal insufficiency, and eosinophilia were identified as independent factors contributing to prolonged survival. Few studies have evaluated the risk factors for the incidence of AEs or specific AEs associated with survival following treatment with nivolumab plus ipilimumab, the efficacy of nivolumab plus ipilimumab in patients with NSCLC in real clinical settings, and the reproducibility of phase 3 clinical trials [14]. The PFS of 6.6 months and ORR of 33.7% observed in the present study, which were obtained using real clinical data (including those of patients with advanced age, comorbidities, and poor PS), were not inferior to those reported in the CheckMate 227 trials [10]. The survival of patients with PD‐L1 < 1% was non‐inferior to that of the patients with PD‐L1 1%–49% (Figure 1c–e). The combination of chemotherapy and PD‐(L)1 was insufficient to treat PD‐L1 TPS < 1%; however, this regimen of anti‐PD‐1 plus anti‐CTLA‐4 antibody may be beneficial for patients with TPS < 1%.

The present study elucidated the details of AEs, including the factors associated with their incidence and their relationship with treatment response. AEs of all grades occurred in 85.3% of patients in the present study; AEs of grade 3 and higher occurred in 39.1%, which is slightly higher than the frequency of AEs reported in a phase III randomized controlled trial [9]. However, only one death due to pneumonia was observed in the present study. No deaths due to sepsis or cytokine release syndrome were observed in the present study, different from the prospective NIPPON study, a randomized phase III trial conducted in Japan that compared the efficacy of nivolumab plus ipilimumab with chemotherapy versus that of pembrolizumab with chemotherapy in patients with NSCLC [34, 35]. A significant difference has been observed in the use of concomitant cytotoxic anticancer drugs. Thrombocytopenia and fever may have been precursors or associated signs of cytokine release syndrome that did not lead to fatal events in the present study. Comparing the results of this retrospective study with those of other trials is difficult; however, abnormal vital signs and blood counts require immediate action, comprising careful and brief observation, hospitalization, and steroid administration. It is undisputed that the combination of nivolumab and ipilimumab, with or without cytotoxic agents, increases the frequency and severity of AEs. Extreme caution is warranted due to the occurrence of rare AEs [36].

The present study aimed to identify the patient background characteristics associated with the incidence of AEs. High PD‐L1 expression was identified as an independent factor for the incidence of all grades of AEs and severe AEs in the multivariate analysis. This finding was consistent with the results of scatter reports that high PD‐L1 expression was associated with the incidence of irAEs following PD‐(L)1 antibody monotherapy [37]. However, clinical trials revealed no correlation between the incidence of irAEs and PD‐L1 expression following PD‐(L)1 antibody monotherapy [38, 39]. A retrospective study of first‐line pembrolizumab in patients with high PD‐L1 expression conducted by Cortellini et al. revealed that the incidence of AEs was correlated with a favorable response rate and PFS. Furthermore, patients with multiple AEs had a better OS than those with a single AE [40]. High PD‐L1 expression may be a predictor of the incidence of AEs associated with treatment responsiveness, considering the mechanisms of AE occurrence, such as shared cancer antigens, potential autoimmune disease, and cytokine activity. Thus, AEs were probable in patients who respond well to treatment (Figure 2e,f and Figure S2). Notably, among the patients treated with nivolumab plus ipilimumab, those who experienced multiple AEs were more likely to survive than those who experienced a single AE [41].

Skin disorders were the most common AEs, followed by liver dysfunction, thyroid dysfunction, and pneumonitis. This finding is similar to those of a phase III study. Compared to that observed in the CheckMate227 study, all grades of hepatitis (29.9%), pneumonia (19.6%), adrenal insufficiency (17.4%), and renal dysfunction (15.2%) were common; however, the analysis of the frequency of incidence of AEs of grade 3 and higher revealed that the incidence of liver dysfunction was almost identical, whereas no incidences of renal dysfunction were reported [9]. Colitis and adrenal insufficiency, which were observed more frequently among patients who experienced AEs of grade 3 and higher, were distinctive findings of the anti‐CTLA‐4 antibody. Pneumonitis (10.9%) was more common among patients who experienced AEs of grade 3 and higher, as well as those who experienced AEs of all grades. This may be attributed in part to the inclusion of patients with interstitial pneumonia complications. Lung injury was the most alarming irAE, and death was observed. Grade 1 eosinophilia was also observed in 9.2% of patients, with a clear correlation with efficacy being observed in terms of survival. Eosinophils, detected in previous studies on anti‐PD‐1 antibodies, may be a candidate prognostic marker in nivolumab plus ipilimumab [42, 43, 44]. Skin disorders and adrenal insufficiency were identified as irAEs that prolonged PFS and OS; they were also identified as independent factors in the multivariate analysis. However, pneumonitis, liver dysfunction, and biliary system abnormalities exhibited no such correlations. Among the putative mechanisms of irAEs, such as the accumulation of existing host autoantibody levels and elevated proinflammatory cytokine levels, increased complement‐mediated inflammation owing to the direct binding of anti‐CTLA‐4 antibodies to CTLA‐4 present in healthy tissues was probably correlated with long survival [45, 46]. The incidence of irAEs suggests that the host immune system is activated. Patients who respond to ICI are treated for a longer duration, which naturally leads to a longer period of exposure to the drug. However, not all background factors are associated with efficacy, and the occurrence of irAEs may be inextricably linked. The frequency of irAEs is correlated with the response rate and survival time when an anti‐PD‐1 antibody is used alone or in combination with chemotherapy [22, 47]. This is the first study to specifically address the effect of AEs and the response to treatment with nivolumab plus ipilimumab. Notably, patients with AEs of all grades exhibited significantly longer survival than those without AEs.

This study has a few limitations. First, variability existed in the timing of each attending physician's judgment of the response and in their assessment of the severity and frequency of toxicity as this was not a prospective study. Second, a bias in relation to efficacy may have been introduced, and the overall number of cases must be increased. Patients who respond to ICI are likely to be treated for a longer duration, which naturally leads to a longer period of exposure to the drug. To minimize immortality time bias, a landmark analysis at 6 weeks was performed, with similar results (Figure S3). Furthermore, validation cohorts and data on the generalisability are lacking. Due to the lack of detailed data on irAE occurrence dates, we did not include a time axis. Consequently, as shown in Table 4, we analyzed the factors influencing the occurrence of irAEs. Third, although the disclosure was made with a minimum observation period of 1 year, the long‐term prognosis was not evaluated. Long‐term observational studies and consideration of biomarkers are planned for the near future [48].

## 5 Conclusions

Patients who experienced any AEs had longer survival than those who did not. In particular, the AEs, PFS, and OS were independently longer when skin disorders, adrenal insufficiency, and eosinophilia occurred.

## Author Contributions

A.M.: data curation, formal analysis, visualization, and writing – original draft. H.I.: conceptualization, methodology, data curation, formal analysis, visualization, supervision, and writing – review and editing. K.K.: conceptualization, methodology, data curation, formal analysis, visualization, supervision, and writing – review and editing. H.K.: conceptualization, methodology, supervision, and writing – review and editing. A.M., H.I., K.K., O.Y., S.E., J.N., K.T., Y.K., K.M., K.H., Y.M., A.S., and K.K.: data curation. All authors contributed to the article and approved the submitted version.

## Disclosure

A.M. receives personal fees from Chugai Pharmaceutical, Ono Pharmaceutical Co. Ltd., Bristol‐Myers Squibb, and AstraZeneca for work outside of the submitted study. O.Y. receives personal fees from Chugai Pharmaceutical, Ono Pharmaceutical Co. Ltd., and Bristol‐Myers Squibb for work outside of the submitted study. K.K. receives personal fees from Ono Pharmaceutical Co. Ltd., Chugai Pharmaceutical, AstraZeneca, Boehringer Ingelheim, Bristol‐Myers Squibb, Pfizer Inc., Kyowa Kirin Co., and Takeda Pharmaceutical for work outside of the submitted study. H.K. receives personal fees from Ono Pharmaceutical Co. Ltd., Chugai Pharmaceutical, AstraZeneca, Boehringer Ingelheim, and Bristol‐Myers Squibb for work outside of the submitted study.

## Ethics Statement

This study was reviewed and approved (2023‐080) by the Institutional Review Board of the Saitama Medical University International Medical Center.

## Conflicts of Interest

The authors declare no conflicts of interest.

## Supporting information


Table S1.

**Figure S1.** Percentage of overall responses and responses according to the PD‐L1 tumor proportion score staining percentage (a) and the incidence of adverse events (b).

**Figure S2.** Comparison between the Kaplan–Meier curves for PFS (a and c) and OS (b and d) of the patient groups with ≥ 3 adverse events, the patient group with ≤ 2 adverse events and the patient group without adverse events, and the patient group with ≥ 4 adverse events, and the patient group with ≤ 3 adverse events and the patient group without adverse events.

**Figure S3.** Comparison between the Kaplan–Meier curves with 6‐week landmark analysis for PFS (a) and OS (b) in patients with or without adverse events. Comparison between the Kaplan–Meier curves with 6‐week landmark analysis for PFS (c) and OS (d) in patients with or without adverse events of grade3 or higher.

**Figure S4.** Subset analysis by smoking status. Comparison of PFS (a) and OS (b) in patients with and without all grade adverse events in patients with smoking. PFS (c) and OS (d) for patients with and without all grade adverse events in patients without smoking.

## Data Availability

Data presented in this study are included in the article/Supporting Information. Further data should be accessed via email to the authors.
