# Supplementary material for: A Real‐Word Analysis of the Correlation Between Clinical Efficacy and Predictive Factors of Immune‐Related Adverse Events in Patients With Nonsmall Lung Cancer Treated With Nivolumab Plus Ipilimumab
Source: Cancer Med. 2025 Apr 18;14(8):e70741. doi: 10.1002/cam4.70741 (PMC12007460; doi:10.1002/cam4.70741)
Supplement: Supplementary file 1 — Table S1. [file CAM4-14-e70741-s003.docx]

Supplementary table 1. Tumor response

|  | All  n, 184 | TPS ≧50%  n, 18 | TPS 1-49%  n, 71 | TPS <1%  n, 84 | Unknown  n, 11 |
| --- | --- | --- | --- | --- | --- |
| CR | 1 | 0 | 0 | 1 | 0 |
| PR | 58 | 9 | 21 | 27 | 1 |
| SD | 61 | 4 | 28 | 26 | 3 |
| PD | 55 | 5 | 17 | 27 | 6 |
| NE | 9 | 0 | 5 | 3 | 1 |
| ORR  (95%CI) | 32.1%  (27.3-40.8) | 50%  (29.0-71.0) | 29.6%  (20.2-41.1) | 33.3%  (22.0-41.5) | 9.1% |
| DCR  (95%CI) | 65.2%  (58.6-72.2) | 72.2%  (45.6-83.9) | 69.0%  (56.0-77.4) | 64.3%  (51.2-71.6) | 36.4% |

TPS, Tumor portion score; CR, complete response; PR, partial response; SD, stable disease; PD progression disease; NE, not evaluation; ORR, overall response rate; CI, confidence interval; DCR, disease control rate

Supplementary table 2. Univariate and multivariate analyses of predictive factors related to (a) PFS and (b) OS

| Factor |  | 1. PFS | Univariate | | | Multivariate | | | (b) OS | Univariate | | | Multivariate | | |
| --- | --- | --- | --- | --- | --- | --- | --- | --- | --- | --- | --- | --- | --- | --- | --- |
|  |  | Median PFS | HR | 95% CI | *p* value | HR | 95% CI | *p* value | Median  OS | HR | 95% CI | *p* value | HR | 95% CI | *p* value |
| Age | ≧75 / <75 | 7.1 / 6.1 | 0.86 | 0.59-1.24 | 0.44 |  |  |  | 12.4 / 17.5 | 1.07 | 0.69-1.62 | 0.76 |  |  |  |
| Sex | Male / Female | 6.7 / 5.4 | 0.87 | 0.59-1.33 | 0.50 |  |  |  | 17.6 / 13.2 | 0.86 | 0.54-1.42 | 0.53 |  |  |  |
| Smoking history | Yes / No | 6.6 / 5.7 | 0.98 | 0.60-1.72 | 0.95 |  |  |  | 17.5 / 13.2 | 0.94 | 0.53-1.80 | 0.83 |  |  |  |
| ECOG-PS | 0-1 / 2 | 7 / 3.1 | 0.63 | 0.41-0.99 | 0.04* | 1.25 | 0.74-2.04 | 0.40 | 19.3 / 4.3 | 0.31 | 0.20-0.51 | <0.01*** | 0.57 | 0.21-1.75 | 0.31 |
| Histology | Sq / Non-Sq | 6.6 / 6.6 | 1.0 | 0.70-1.45 | 0.92 |  |  |  | 14.7 / 17.6 | 1.11 | 0.73-1.66 | 0.62 |  |  |  |
| Stage | Rec post-surgery / Ⅲ・Ⅳ | 7.9 / 5.9 | 0.80 | 0.50-1.21 | 0.29 |  |  |  | 29.4 / 12.6 | 0.48 | 0.25-0.81 | 0.01* |  |  |  |
|  | Rec post-curative radiation / Ⅲ・Ⅳ | 3.3 / 6.8 | 1.50 | 0.90-2.38 | 0.10 |  |  |  | 17.5 / 12.8 | 1.34 | 0.76-2.21 | 0.28 |  |  |  |
| PD-L1 | ≧1 / < 1 | 6.9 / 5.6 | 0.38 | 0.62-1.20 | 0.38 |  |  |  | 18.2 / 12.8 | 0.77 | 0.53-1.14 | 0.19 |  |  |  |
|  | ≧50 / < 50 | 9.2 / 6.5 | 0.45 | 0.21-0.84 | 0.02* | 0.35 | 0.16-0.66 | <0.01*** | NR / 14 | 0.54 | 0.24-1.04 | 0.09 | 0.42 | 0.17-0.91 | 0.03* |
| BMI | ≧22 / <22 | 6.7 / 6.1 | 0.96 | 0.69-1.33 | 0.79 |  |  |  | 19.1 / 13 | 0.72 | 0.49-1.06 | 0.09 |  |  |  |
| GPS | 0-1 / 2 | 8.5 / 3.1 | 0.55 | 0.39-0.80 | <0.01*** | 0.56 | 0.38-0.84 | <0.01*** | 24.4 / 5.2 | 0.31 | 0.21-0.46 | <0.01*** | 0.93 | 0.45-2.06 | 0.84 |
| NLR | ≧5 / <5 | 4.2 / 6.9 | 1.32 | 0.93-1.84 | 0.11 |  |  |  | 9.4 / 23.5 | 1.81 | 1.22-2.65 | <0.01** | 0.92 | 0.50-1.61 | 0.79 |
| Brain metastasis | Yes / No | 9.5 / 6.1 | 0.75 | 0.46-1.17 | 0.22 |  |  |  | NR / 13.2 | 0.47 | 0.24-0.84 | 0.02* |  |  |  |
| Liver metastasis | Yes / No | 3.2 / 6.8 | 1.82 | 1.07-2.90 | 0.02* | 1.69 | 0.99-2.74 | 0.06 | 8.9 / 18.0 | 1.99 | 1.12-3.29 | 0.01* | 1.05 | 0.31-2.59 | 0.93 |
| Bone metastasis | Yes / No | 6.9 / 5.1 | 1.41 | 0.99-1.97 | 0.05* | 1.35 | 0.95-1.90 | 0.10 | 11 / 18.2 | 1.43 | 0.96-2.13 | 0.07 |  |  |  |
| Malignant effusion | Yes / No | 4.8 / 6.8 | 1.20 | 0.81-1.73 | 0.34 |  |  |  | 9.7 / 18.5 | 1.01 | 0.57-1.83 | 0.05* |  |  |  |

HR, hazard ratio; CI, confidence interval; ECOG-PS, Eastern Cooperative Oncology Group performance status; Sq, squamous carcinoma; Rec, recurrence; PD-L1, programmed death-ligand 1; BMI, body mass index; GPS, Glasgow prognostic score; NLR, neutrophil-to-lymphocyte ratio; Cr, creatinine

*p＜0.05

**p<0.01

***p<0.001

Supplementary table 3. Comparison of tumor response and progression-free survival between groups with and without adverse event

| Efficacy |  | Total  n, 184 (%) | AE  N=154 (%) | No AE  N=30 (%) | *p* value | ≧G3 AE  N=72 (%) | No ≧G3 AE  N=112 (%) | *p* value |
| --- | --- | --- | --- | --- | --- | --- | --- | --- |
| Best Response | CR/PR  SD/PD  NE | 59 (32.1)  116 (63.0)  9 (4.9) | 57 (37.0)  92 (59.7)  5 (3.2) | 2 (6.7)  24 (80)  4 (13.3) | 0.02* | 32 (44.4)  40 (55.5) | 27 (24.1)  76 (67.8)  NE 9 | 0.01* |
|  | CR/PR/SD  PD | 120 (65.2)  55 (29.9) | 110 (71.4)  39 (25.3)  NE 5 | 10 (33.3)  16 (53.3)  NE 4 | <0.01*** | 56 (77.8)  16 (22.2) | 64 (57.1)  39 (34.8)  NE 9 | 0.03* |
| PFS | <6m  ≧6m | 90 (48.9)  94 (51.1) | 64 (41.6)  90 (58.4) | 26 (86.7)  4 (13.3) | <0.01*** | 29 (40.3)  43 (59.7) | 61 (54.5)  51 (45.5) | 0.07 |
|  | <12m  ≧12m | 132 (71.7)  52 (28.2) | 104 (67.5)  50 (32.5) | 28 (93.3)  2 (6.7) | <0.01** | 45 (62.5)  27 (37.5) | 87 (77.7)  25 (22.3) | 0.03* |

AE, adverse event; G3, grade3; CR, complete response; PR, partial response; SD, stable disease; PD progression disease; NE, not evaluation; PFS, progression-free survival; m, months

*p＜0.05

**p<0.01

***p<0.001
