# Supplementary figures and images for: A Real‐Word Analysis of the Correlation Between Clinical Efficacy and Predictive Factors of Immune‐Related Adverse Events in Patients With Nonsmall Lung Cancer Treated With Nivolumab Plus Ipilimumab
Source: Cancer Med. 2025 Apr 18;14(8):e70741. doi: 10.1002/cam4.70741 (PMC12007460; doi:10.1002/cam4.70741)

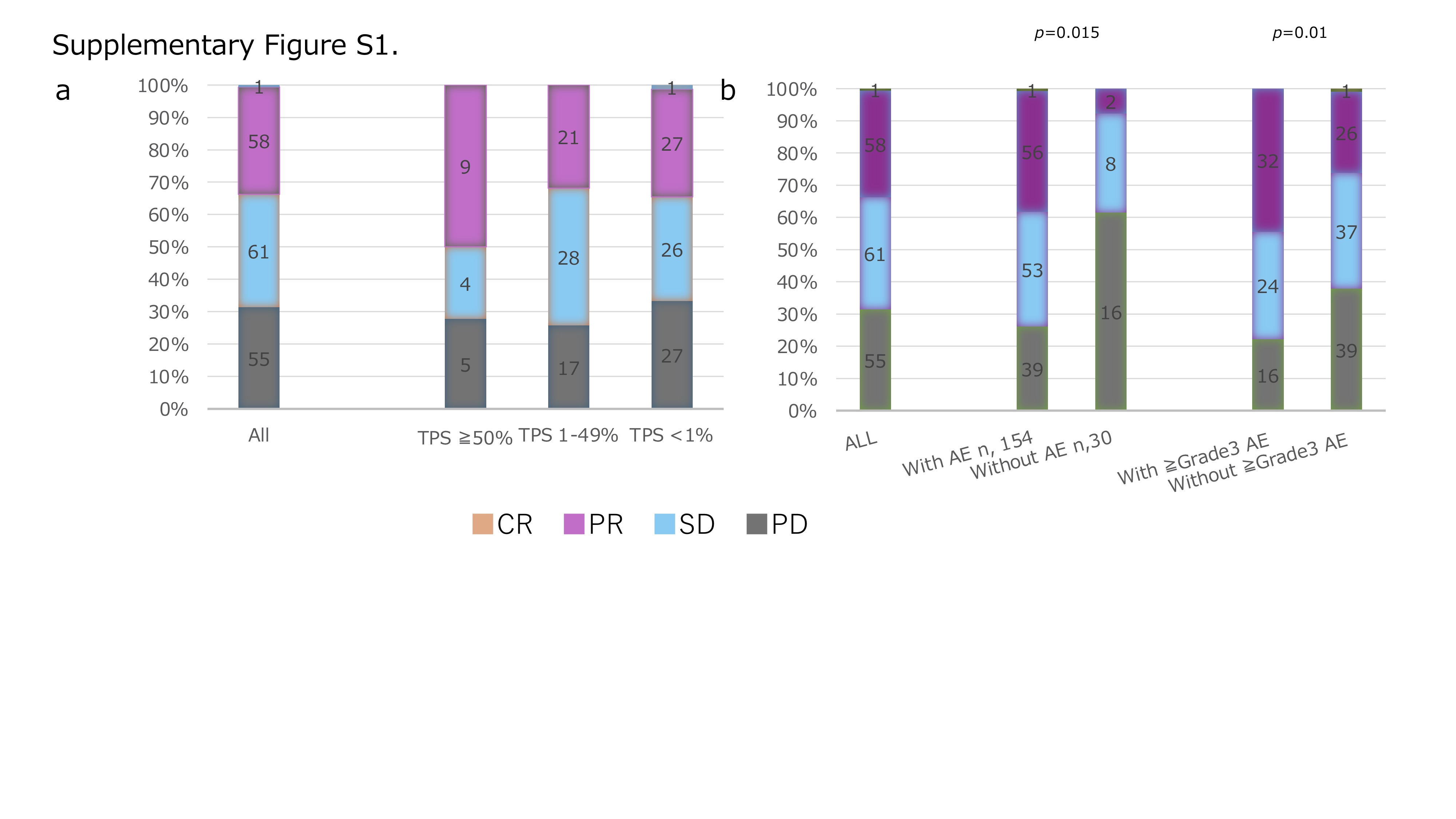

Supplement: Supplementary file 2 — Figure S1. Percentage of overall responses and responses according to the PD‐L1 tumor proportion score staining percentage (a) and the incidence of adverse events (b). [file CAM4-14-e70741-s004.jpg]

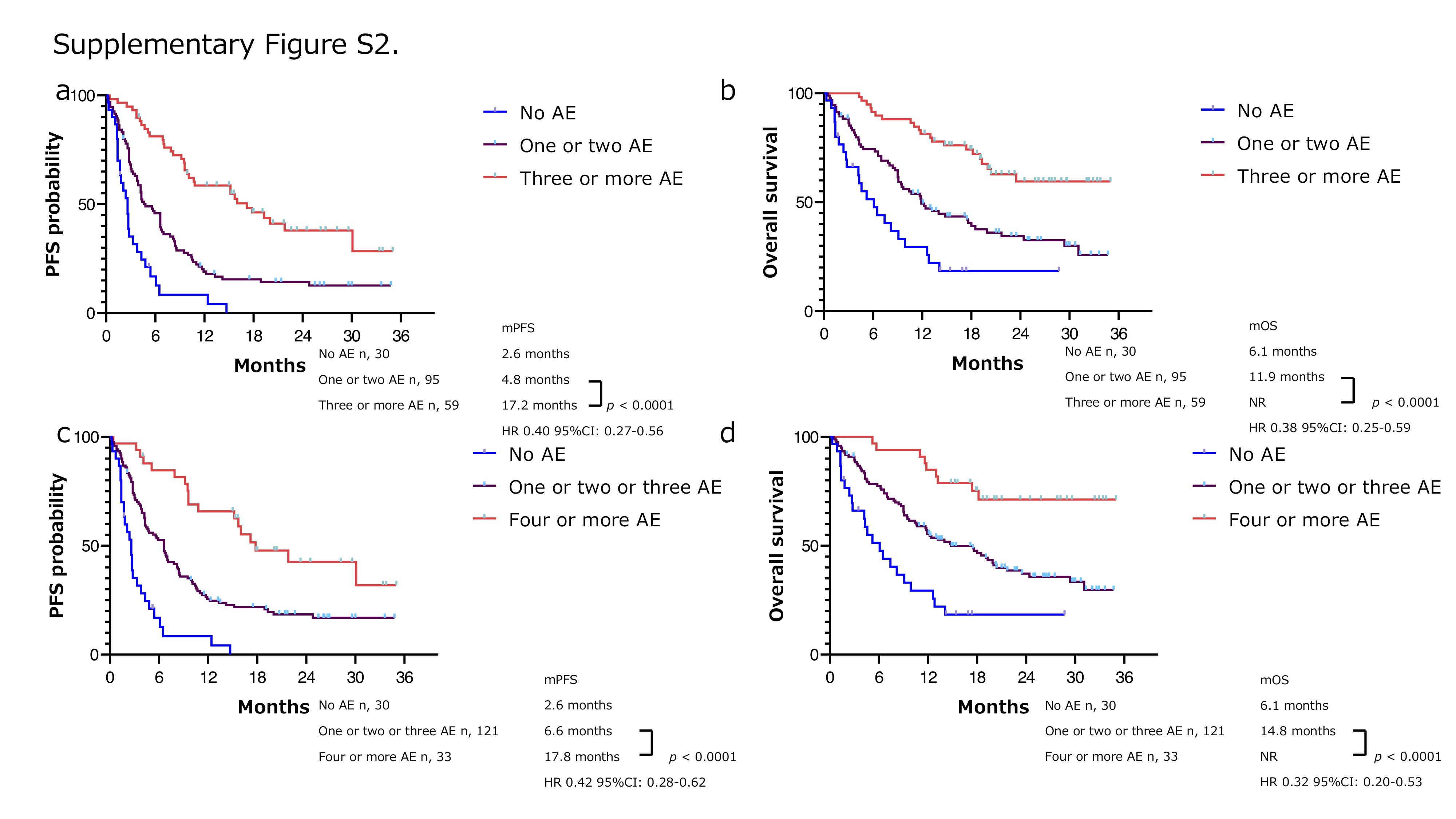

Supplement: Supplementary file 3 — Figure S2. Comparison between the Kaplan–Meier curves for PFS (a and c) and OS (b and d) of the patient groups with ≥ 3 adverse events, the patient group with ≤ 2 adverse events and the patient group without adverse events, and the patient group with ≥ 4 adverse events, and the patient group with ≤ 3 adverse events and the patient group without adverse events. [file CAM4-14-e70741-s001.jpg]

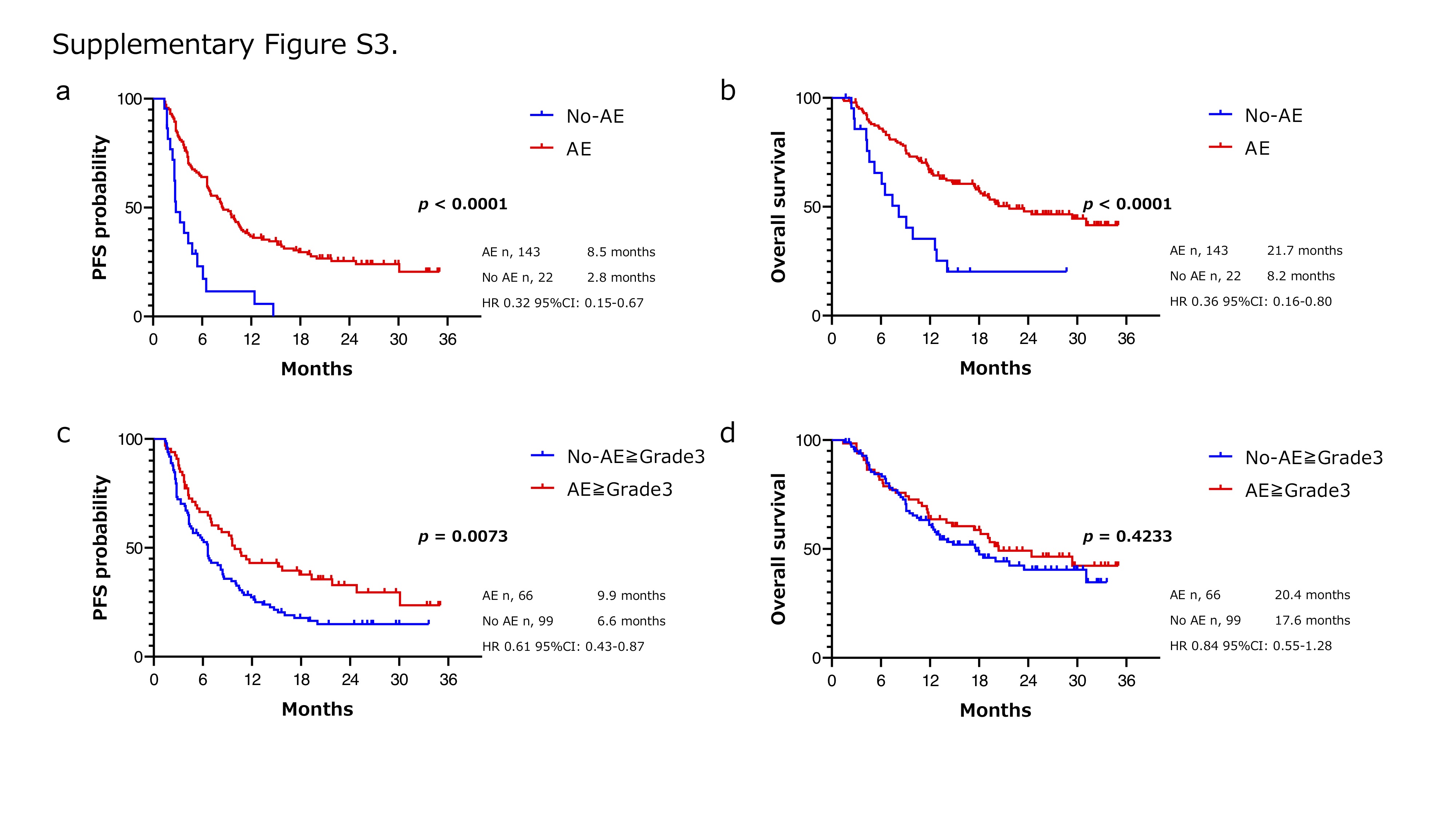

Supplement: Supplementary file 4 — Figure S3. Comparison between the Kaplan–Meier curves with 6‐week landmark analysis for PFS (a) and OS (b) in patients with or without adverse events. Comparison between the Kaplan–Meier curves with 6‐week landmark analysis for PFS (c) and OS (d) in patients with or without adverse events of grade3 or higher. [file CAM4-14-e70741-s002.jpg]

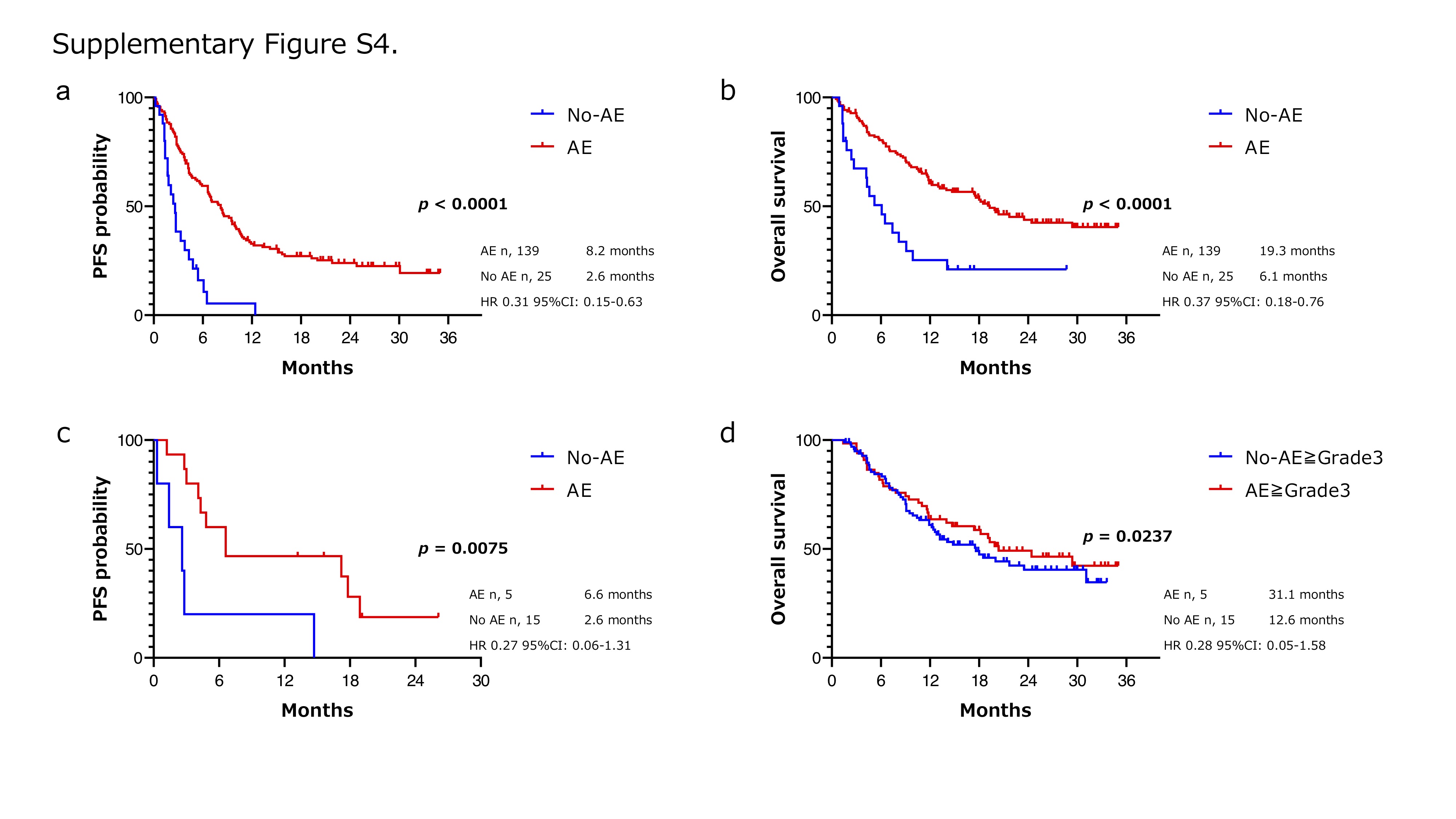

Supplement: Supplementary file 5 — Figure S4. Subset analysis by smoking status. Comparison of PFS (a) and OS (b) in patients with and without all grade adverse events in patients with smoking. PFS (c) and OS (d) for patients with and without all grade adverse events in patients without smoking. [file CAM4-14-e70741-s005.jpg]
